# Aspartate aminotransferase to alanine aminotransferase ratio is associated with frailty and mortality in older patients with heart failure

**DOI:** 10.1038/s41598-021-91368-z

**Published:** 2021-06-07

**Authors:** Daichi Maeda, Nobuyuki Kagiyama, Kentaro Jujo, Kazuya Saito, Kentaro Kamiya, Hiroshi Saito, Yuki Ogasahara, Emi Maekawa, Masaaki Konishi, Takeshi Kitai, Kentaro Iwata, Hiroshi Wada, Masaru Hiki, Taishi Dotare, Tsutomu Sunayama, Takatoshi Kasai, Hirofumi Nagamatsu, Tetsuya Ozawa, Katsuya Izawa, Shuhei Yamamoto, Naoki Aizawa, Ryusuke Yonezawa, Kazuhiro Oka, Shin-ichi Momomura, Yuya Matsue

**Affiliations:** 1 Department of Cardiology, Osaka Medical College, Takatsuki, Japan; 2 Department of Cardiology, The Sakakibara Heart Institute of Okayama, Okayama, Japan; 3 Department of Digital Health and Telemedicine R&D, Juntendo University, Tokyo, Japan; 4 Department of Cardiovascular Biology and Medicine, Faculty of Medicine, Juntendo University, Tokyo, Japan; 5 Department of Cardiology, Nishiarai Heart Center Hospital, Tokyo, Japan; 6 Department of Rehabilitation, The Sakakibara Heart Institute of Okayama, Okayama, Japan; 7 Department of Rehabilitation, School of Allied Health Sciences, Kitasato University, Sagamihara, Japan; 8 Department of Rehabilitation, Kameda Medical Center, Kamogawa, Japan; 9 Department of Cardiovascular Biology and Medicine, Juntendo University Graduate School of Medicine, Tokyo, Japan; 10 Department of Nursing, The Sakakibara Heart Institute of Okayama, Okayama, Japan; 11 Department of Cardiovascular Medicine, Kitasato University School of Medicine, Sagamihara, Japan; 12 Division of Cardiology, Yokohama City University Medical Center, Yokohama, Japan; 13 Department of Cardiovascular Medicine, Kobe City Medical Center General Hospital, Kobe, Japan; 14 Department of Rehabilitation, Kobe City Medical Center General Hospital, Kobe, Japan; 15 Department of Cardiovascular Medicine, Saitama Medical Center, Jichi Medical University, Saitama, Japan; 16 Cardiovascular Respiratory Sleep Medicine, Juntendo University Graduate School of Medicine, Tokyo, Japan; 17 Department of Cardiology, Tokai University School of Medicine, Isehara, Japan; 18 Department of Rehabilitation, Odawara Municipal Hospital, Odawara, Japan; 19 Department of Rehabilitation, Kasukabe Chuo General Hospital, Kasukabe, Japan; 20 Department of Rehabilitation, Shinshu University Hospital, Matsumoto, Japan; 21 Department of Cardiovascular Medicine, Nephrology and Neurology, University of the Ryukyus, Okinawa, Japan; 22 Rehabilitation Center, Kitasato University Medical Center, Kitamoto, Japan; 23 Department of Rehabilitation, Saitama Citizens Medical Center, Saitama, Japan; 24 Saitama Citizens Medical Center, Saitama, Japan

**Keywords:** Heart failure, Prognostic markers, Geriatrics

## Abstract

Frailty is a common comorbidity associated with adverse events in patients with heart failure, and early recognition is key to improving its management. We hypothesized that the AST to ALT ratio (AAR) could be a marker of frailty in patients with heart failure. Data from the FRAGILE-HF study were analyzed. A total of 1327 patients aged ≥ 65 years hospitalized with heart failure were categorized into three groups based on their AAR at discharge: low AAR (AAR < 1.16, n = 434); middle AAR (1.16 ≤ AAR < 1.70, n = 487); high AAR (AAR ≥ 1.70, n = 406). The primary endpoint was one-year mortality. The association between AAR and physical function was also assessed. High AAR was associated with lower short physical performance battery and shorter 6-min walk distance, and these associations were independent of age and sex. Logistic regression analysis revealed that high AAR was an independent marker of physical frailty after adjustment for age, sex and body mass index. During follow-up, all-cause death occurred in 161 patients. After adjusting for confounding factors, high AAR was associated with all-cause death (low AAR vs. high AAR, hazard ratio: 1.57, 95% confidence interval, 1.02–2.42; *P* = 0.040). In conclusion, AAR is a marker of frailty and prognostic for all-cause mortality in older patients with heart failure.

## Introduction

Frailty is a geriatric syndrome characterized by a decline in functional reserve and increased vulnerability to stressors^1^. Frailty is common and associated with worse clinical outcomes in patients with acute^2^ and chronic heart failure^3,4^. Since frailty is reversible and patients can possibly return to a healthy state, early identification is essential. However, simple and readily available markers for frailty are yet to be found.

We previously demonstrated a significant association between high aspartate aminotransferase (AST) to alanine aminotransferase (ALT) ratio (AAR) and low body mass index, malnutrition, and worse outcomes in patients with acute heart failure^5^, suggesting high AAR as a marker of frailty status in patients with heart failure. However, the direct association between AAR and physical frailty or exercise capacity has not been evaluated in older patients with heart failure. Therefore, we aimed to evaluate the association between AAR and physical frailty, exercise capacity, and prognosis in older patients with heart failure.

## Results

**Figure 1:**
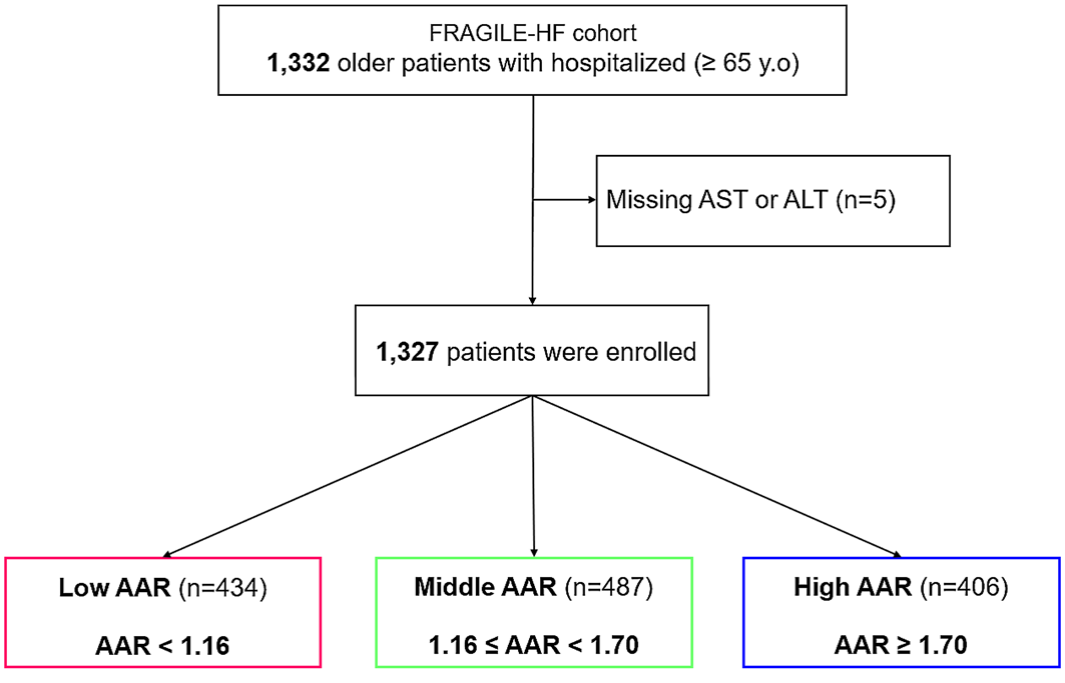
Study flowchart: Participants were divided into three groups based on their aspartate aminotransferase to alanine aminotransferase ratio. *AAR* aspartate aminotransferase to alanine aminotransferase ratio, *ALT* alanine aminotransferase, *AST* aspartate aminotransferase, *HF* heart failure.

During our study period, 1332 hospitalized heart failure patients aged ≥ 65 years were registered in the FRAGILE-HF study. Patients lacking AST or ALT values (n = 5) were excluded; thus, a total of 1327 patients (age: 80.2 ± 7.8 years, 43% females) were enrolled in the analysis. Patients were assigned into three groups according to the AAR cut-off values: low AAR, AAR < 1.16 (n = 434); middle AAR, 1.16 ≤ AAR < 1.70 (n = 487); and high AAR, AAR ≥ 1.70 (n = 406) (Fig. 1). The baseline characteristics are described in Table 1. Patients with high AAR were older in age, more of females, and had New York Heart Association class III/IV, prior history of heart failure and use of loop diuretics, lower body mass index, and higher left ventricular ejection fraction. Laboratory data showed that patients with high AAR had higher brain natriuretic peptide (BNP) and lower albumin, AST, ALT, estimated glomerular filtration rate, and hemoglobin levels.

**Table 1:** Baseline clinical characteristics of the patients.

	AAR tertile			*P* value
	Low AAR	Middle AAR	High AAR	
	n = 434	n = 487	n = 406	
AAR at discharge	0.92 [0.79–1.05]	1.40 [1.29–1.53]	2.00 [1.86–2.33]	<0.001
**Clinical demographics**				
Age, years	78 [71–84]	81 [74–86]	84 [78–88]	<0.001
Male, n (%)	320 (73.7%)	278 (57.1%)	159 (39.2%)	<0.001
Body mass index, kg/m²	21.4 [19.6–23.7]	21.2 [19.1–23.7]	20.0 [17.8–22.1]	<0.001
Current smoker, n (%)	58 (13.4%)	46 (9.4%)	25 (6.2%)	0.002
NHYA class III/IV, n (%)	48 (11.1%)	71 (14.6%)	78 (19.2%)	0.004
Systolic blood pressure, mmHg	112 [102–125]	112 [102–126]	112 [102–124]	0.529
Diastolic blood pressure, mmHg	62 [56–68]	61 [56–70]	60 [54–68]	0.031
Heart rate, bpm	70 [60–79]	70 [60–79]	70 [61–80]	0.235
Left ventricular ejection fraction, %	40 [31–58]	45 [32–60]	50 [34–62]	<0.001
Prior history of heart failure, n (%)	145 (33.4%)	204 (41.9%)	200 (49.3%)	<0.001
Hypertension, n (%)	312 (71.9%)	352 (72.3%)	281 (69.2%)	0.560
Dyslipidemia, n (%)	162 (37.3%)	189 (38.8%)	123 (30.3%)	0.021
Diabetes mellitus, n (%)	156 (35.9%)	199 (40.9%)	119 (29.3%)	0.002
Coronary artery disease, n (%)	153 (35.3%)	173 (35.5%)	142 (35.0%)	0.986
COPD, n (%)	53 (12.2%)	49 (10.1%)	41 (10.1%)	0.501
**Laboratory data **				
Albumin, g/dL	3.6 [3.2–3.8]	3.5 [3.2–3.8]	3.3 [3.0–3.6]	<0.001
Blood urea nitrogen, mg/dL	25 [19–34]	25 [20–35]	28 [20–38]	0.026
Creatinine, mg/dL	1.12 [0.90–1.44]	1.16 [0.93–1.60]	1.23 [0.92–1.69]	0.008
eGFR, mL/min/1.73m²	60 [44–74]	52 [36–69]	46 [31–62]	<0.001
Aspartate aminotransferase, U/L	25 [19–32]	22 [18–28]	21 [17–27]	<0.001
Alanine aminotransferase, U/L	28 [20–40]	16 [12–20]	10 [8–13]	<0.001
Sodium, mEq/L	139 [137–141]	139 [137–141]	140 [137–142]	0.099
Hemoglobin, g/dL	12.5 [11.2–14.0]	11.7 [10.4–13.0]	10.7 [9.6–12.0]	<0.001
Brain natriuretic peptide, pg/mL	231.6 [118.7–451.2]	271.8 [130.1–477.8]	331.8 [162.0–601.5]	<0.001
**Medication **				
ACE-I/ARB, n (%)	304 (70.0%)	338 (69.4%)	249 (61.3%)	0.011
β-blockers, n (%)	337 (77.6%)	376 (77.2%)	253 (62.3%)	<0.001
Loop diuretics, n (%)	359 (82.7%)	435 (89.3%)	364 (89.7%)	0.002
MRA, n (%)	242 (55.8%)	240 (49.3%)	178 (43.8%)	0.003

Values are presented as number (%) or median [interquartile range].

AAR, aspartate aminotransferase to alanine aminotransferase ratio; NYHA, New York Heart Associations; COPD, chronic obstructive pulmonary disease; eGFR, estimated glomerular filtration rate; ACE-I, angiotensin-converting-enzyme inhibitor; ARB, angiotensin II receptor blocker; MRA, mineralocorticoid receptor antagonists.

Regarding frailty and nutritional status, univariable and multivariable linear regression analysis revealed that handgrip strength, 6-min walk distance, short physical performance battery (SPPB), and geriatric nutritional risk index (GNRI) were significantly lower in patients with AAR ≥ 1.70 compared to those with AAR < 1.16 after adjustment for age and sex (Table 2). Univariate and multivariable logistic regression showed that AAR ≥ 1.70 was significantly associated with physical frailty even after adjustment for age, sex and body mass index (Table 3). We also checked the association between all these factors and AAR as a continuous variable on univariate/multivariable linear regression analysis and found that these findings were not significantly changed (handgrip strength, t value − 4.69, *P* < 0.001; 6-min walk distance, t value − 2.04, *P* = 0.041; SPPB, t value − 1.70, *P* = 0.089; GNRI, t value − 4.36, *P* < 0.001).

**Table 2:** Association between AAR and physical status.

	Unadjusted model					Adjusted model*					
	Coefficient	95% CI	Standard error	t value	*P* value	Coefficient	95% CI	Standard error	t value	R²	*P* value
**Hand grip strength**										0.503	
Low vs. middle AAR	− 2.97	− 3.94–− 2.00	0.50	− 5.99	<0.001	− 0.90	− 1.64–− 0.16	0.38	− 2.38		0.017
Low vs. high AAR	− 6.55	− 7.57–− 5.53	0.52	− 12.61	<0.001	− 2.10	− 2.91–− 1.28	0.41	− 5.05		<0.001
**6**− **minute walk distance**										0.243	
Low vs. middle AAR	− 25.01	− 41.63–− 8.38	8.47	− 2.95	0.003	− 3.18	− 18.23–11.87	7.67	− 0.41		0.678
Low vs. high AAR	− 70.37	− 87.99–52.74	8.98	− 7.83	<0.001	− 22.04	− 38.75–− 5.33	8.52	− 2.59		0.010
**SPPB**										0.252	
Low vs. middle AAR	− 0.65	− 1.07–− 0.24	0.21	− 3.12	0.002	− 0.10	− 0.47–0.27	0.19	− 0.52		0.600
Low vs. high AAR	− 1.77	− 2.20–− 1.34	0.22	− 8.06	<0.001	− 0.51	− 0.92–− 0.10	0.21	− 2.46		0.014
**GNRI**										0.093	
Low vs. middle AAR	− 0.93	− 2.34–0.48	0.72	− 1.29	0.197	− 0.35	− 1.74–1.05	0.71	− 0.49		0.624
Low vs. high AAR	− 5.33	− 6.80–− 3.82	0.76	− 7.05	<0.001	− 3.94	− 5.47–− 2.40	0.78	− 5.02		<0.001

*Adjusted for age and gender.

AAR, aspartate aminotransferase to alanine aminotransferase ratio; CI, confidence interval; GNRI, geriatric nutritional risk index; SPPB, short physical performance battery.

**Table 3:** Logistic analysis for physical frailty according to the Fried phenotype model.

	Unadjusted model			Adjusted model*		
	Odds ratio	95% CI	*P* value	Odds ratio	95% CI	*P* value
**AAR as categorical variable**						
Low AAR	1 (reference)			1 (reference)		
Middle AAR	1.37	1.05–1.80	0.022	1.24	0.94–1.64	0.127
High AAR	1.91	1.43–2.56	<0.001	1.45	1.06–1.99	0.021
**AAR as continuous variable **	1.66	1.35–2.05	<0.001	1.39	1.11–1.73	0.004

* Adjusted for age, gender and body mass index.

AAR, aspartate aminotransferase to alanine aminotransferase ratio; CI, confidence interval.

**Figure 2:**
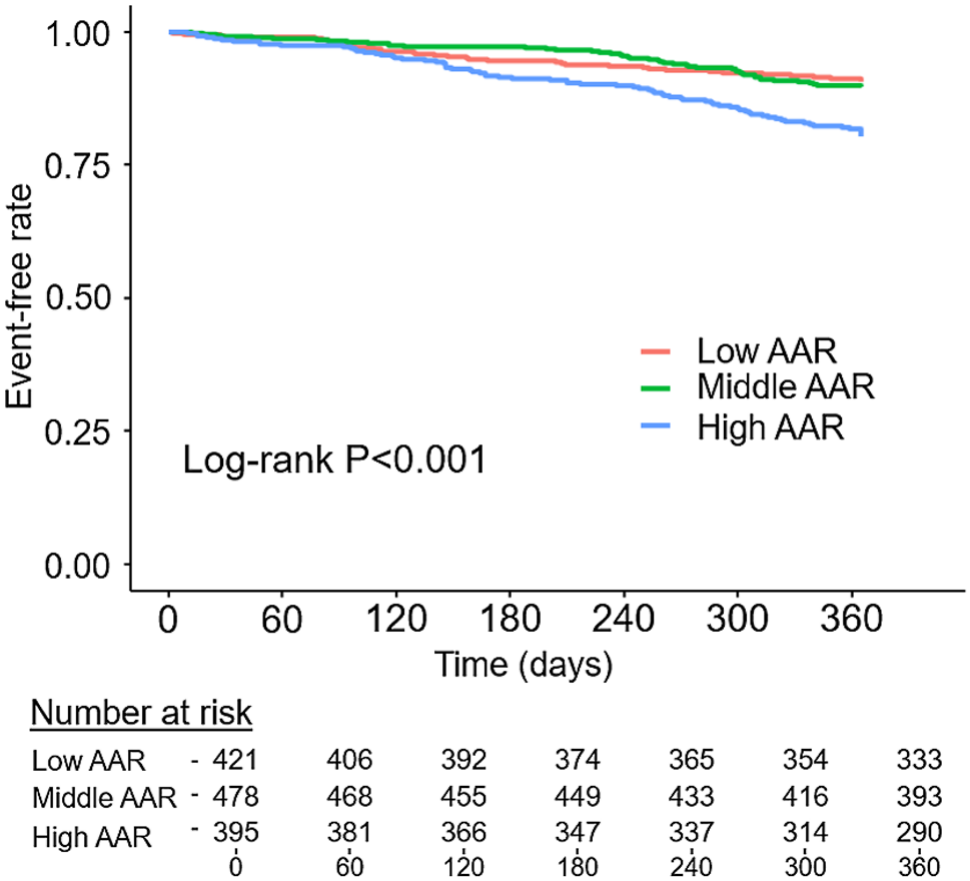
Kaplan–Meier curves of all-cause death stratified by aspartate aminotransferase to alanine aminotransferase ratio. *AAR* aspartate aminotransferase to alanine aminotransferase ratio.

Data regarding 1-year mortality were collected for 97.5% of the 1,327 study patients, and all-cause death occurred in 161 (12.4%) patients. Kaplan–Meier curves showed that event-free rates were lower among patients with high AAR than in the other two groups (Fig. 2, log-rank *P* < 0.001). Cox proportional hazards analysis after excluding 214 patients (16.1%) with missing data on prognosis or adjustment variables showed that patients with high AAR had a significant higher risk of 1-year mortality than those with low AAR even after adjusting for the Meta-analysis Global Group in Chronic Heart Failure (MAGGIC) risk score and log-transformed BNP (Table 4). We also performed multiple imputations by creating 20 imputed datasets as sensitivity analysis and obtained the same results in the adjusted model (hazard ratio: 0.92, 95% CI: 0.60–1.42 for middle AAR, and hazard ratio: 1.51, 95% CI: 1.01–2.26, *P* = 0.043 for high AAR). Moreover, the interaction between AAR and sex was investigated because sex may have significant interaction with frailty. Consequently, we found that there was no significant interaction between AAR and sex (interaction *P* = 0.430).

**Table 4:** Cox proportional hazard analysis for all-cause death.

	Unadjusted model				Adjusted model*			
	C-index	Hazard ratio	95% CI	*P* value	C-index	Hazard ratio	95% CI	*P* value
**Groups **	0.59				0.71			
Low AAR		1 (reference				1 (reference)		
Middle AAR		1.08	0.70–1.67	0.715		0.98	0.62–1.56	0.938
High AAR		2.19	1.48–3.25	<0.001		1.57	1.02–2.42	0.040

* Adjusted for Meta-Analysis Global Group in Chronic Heart Failure risk score and log brain natriuretic peptide.

AAR, aspartate aminotransferase to alanine aminotransferase ratio; CI, confidence interval.

To test if this association was mainly driven by AST alone or ALT alone, we performed the same analysis by segmenting the entire cohort into three groups stratified by the tertile of AST and ALT. For ALT, a higher tertile group was associated with lower handgrip strength and higher GNRI, but not with SPPB and 6-min walk distance after adjustment for age and sex (Supplementary Table 1). Likewise, higher tertile groups of AST were not associated with handgrip strength, 6-min walk distance, SPPB, and GNRI (Supplementary Table 2). We compared the prognostic predictability of AST, ALT, and AAR using the area under the receiver operating characteristic curve (AUC) (AAR: AUC 0.60, 95% confidence interval [CI] 0.56–0.65; AST: AUC 0.51, 95% CI 0.46–0.56; ALT: AUC 0.56, 95% CI 0.51–0.61) and found that AAR exceeded AST and ALT in AUC (AAR vs. AST, *P* = 0.014; AAR vs. ALT, *P* = 0.017). Moreover, neither AST nor ALT was associated with 1-year mortality after adjusting for the MAGGIC risk score and log-transformed BNP (Supplementary Table 3).

## Discussion

In the current study evaluating patients hospitalized for heart failure, we demonstrated that: 1) high AAR was associated with physical frailty, malnutrition, and lower exercise capacity independent of age and sex, 2) high AAR was independently associated with greater 1-year mortality. Our study results imply that AAR can be a potential surrogate marker of frailty in patients with heart failure.

Physical frailty is known as a clinical syndrome composed of five physical conditions, including weakness, slow gait speed, exhaustion, weight loss, and low physical activities^6^. It progresses through a vicious circle of decrease in muscle strength, metabolic rate, energy expenditure, and worsening nutritional status^7^. Thus, decreased physical activity and malnutrition are the important components of physical frailty. In the current study, we showed that high AAR was associated with low SPPB, short 6-min walk distance, poor hand grip strength, and worse nutritional status. Although we demonstrated the association between high AAR and poor prognosis, the mechanism behind the association of AAR and physical function has not been well demonstrated. However, several possible mechanisms have been proposed for the association between high AAR and frailty. For instance, ALT plays an essential role in gluconeogenesis, the process of breaking down muscle protein into amino acids, converting alanine to α-ketoglutarate to produce energy^8^. Moreover, a previous study on a diabetes mellitus cohort reported that low ALT was correlated with low handgrip strength^9^. Given these study results, it could be that ALT is the main driver of the association between higher AAR and frailty; however, as we clearly showed in our study results, AAR was more strongly associated with frailty, exercise capacity, and 1-year mortality independently of other covariates, than ALT alone. This may because the proportion of AST and ALT offsets the impact of liver dysfunction and makes AAR more specific to muscle capacity, which cannot be evaluated otherwise. AAR is thus a potential prognostic predictor independent of other covariates in patients with heart failure.

Frailty may be associated with sex, and a previous study including 10% of patients with heart failure suggested that frailty had a significant interaction with sex on survival^10^. However, we cannot find any interaction between sex and frailty. The discrepancy may be explained by the differences in baseline characteristics. Our cohort was all heart failure patients, and was older and had lower body mass index than a previous cohort. However, we should keep “the sex difference” in mind.

We also reconfirmed the association between AAR and malnutrition, which we have previously described in patients with acute heart failure^5^. Malnutrition often leads to a deficiency of pyridoxal-5′-phosphatase^11^, which is the biologically active form of vitamin B6. ALT is more vulnerable to pyridoxal-5′-phosphatase deprivation than AST^12^, which may explain the association between malnutrition and higher AAR. Indeed, in healthy populations aged ≥ 65 years, lower ALT was associated with lower vitamin B6 levels and physical frailty^13^. Reconfirmation of this association in the FRAGILE-HF cohort with predominantly older patients strengthens our hypothesis; however, it should be further investigated in future studies as GNRI is not a gold standard of nutritional status in patients with heart failure, and pyridoxal-5-phosphatase was not directly evaluated in this study.

In summary, our study results suggest that high AAR may reflect poor physical function and malnutrition, which are important components of the frailty cycle. This implies the possibility of AAR to serve as a marker for screening/monitoring of frailty and subsequent poor prognostic outcomes in older patients with heart failure. As both AST and ALT are readily available and are easily measurable biomarkers in daily clinical practice, early identification of those at a high risk of frailty among older patients with heart failure may lead to early intervention, including nutritional intervention or exercise rehabilitation, and subsequently, a better prognosis. Muscle training and nutritional supplements may be effective for frail patients with heart failure. Aerobic endurance training and resistance training were associated with an improvement in exercise capacity and quality of life in patients with heart failure^14,15^. However, this hypothesis should be evaluated in large-scale randomized controlled trials in the future.

There are several limitations in the current study. First, we could not exclude those with a history of liver disease or heavy consumption of alcohol that can impact liver function tests and subsequently AAR and its association with prognosis. Second, because laboratory data were obtained only at discharge, serial changes in AAR were not available, and we could not analyze their clinical characteristics and prognostic implications. Third, as our current study did not include patients under hemodialysis and those aged < 65 years; whether our study results apply to such patients should be tested in future studies. Fourth, although we performed multivariable analyses adjusting for confounding variables, the results may yet be confounded by other factors. Finally, our study included only Japanese patients, and our cohort (mean body mass index, 21.4 ± 3.8 kg/m^2^) was much smaller physically than a general Western cohort. Therefore, whether the results of our study can apply to Western populations is unknown.

In conclusion, AAR, which is readily available at a low cost, is strongly associated with frailty and poor prognosis in older patients with hospitalized heart failure.

## Methods

### Study design and patient population

This is a post hoc analysis of the FRAGILE-HF cohort study, in which 1,332 hospitalized patients aged ≥ 65 years with decompensation of heart failure, who could ambulate at discharge, were included. The study design and main results have already been published elsewhere^16^. Briefly, the main objective of FRAGILE-HF was to evaluate the prevalence and prognostic impact of multi-frailty domains in older patients with heart failure who require hospitalization. The exclusion criteria were: (1) previous heart transplantation or left ventricular assist device implantation, (2) chronic peritoneal dialysis or hemodialysis, and (3) acute myocarditis. Patients with missing BNP or N-terminal-proBNP data and patients with a BNP level < 100 pg/mL or N-terminal-proBNP level < 300 pg/mL at admission were also excluded as the diagnosis may be unclear in these cases. We enrolled patients with both heart failure with reduced and preserved ejection fraction. Fifteen hospitals in Japan enrolled patients from September 2016 to March 2018. Before discharge, physical examination, echocardiography, blood samples, and drug history were obtained when patients were stable. From the AST and ALT values obtained before discharge, patients were assigned into three groups based on predetermined AAR cut-off values as follows: low AAR, AAR < 1.16; middle AAR, 1.16 ≤ AAR < 1.70; and high AAR, AAR ≥ 1.70^5^.

All participants were notified regarding their participation in the present study, and it was explained that they were free to opt out of participation at any time. Written, informed consent was obtained from each patient before enrolment. Our study complies with the Declaration of Helsinki and the Japanese Ethical Guideline for Medical and Health Research involving Human Subjects. The study protocol was approved by the Sakakibara Heart Institution of Okayama Research Ethics Committee. Study information, including objectives, inclusion and exclusion criteria, primary outcome, and the names of participating hospitals, were published in the publicly available University Hospital Information Network (UMIN-CTR, unique identifier: UMIN000023929) before the first patient was enrolled.

### Assessment of physical frailty and physical function

Physical frailty was defined by the Fried phenotype model, which is the most widely applied model, and generally considered as the standard model for physical frailty^6^. According to Fried's model, the frailty phenotype consists of the following five elements: slowness (gait speed), weakness (handgrip strength), weight loss, exhaustion, and low physical activity^17^. Patients who met ≥ 3 out of 5 criteria were considered physically frail according to previous studies and the predefined definition in FRAGILE-HF^16,18,19^.

We also evaluated SPPB and 6-min walk distance, performed by experienced physical therapists and/or heart failure specialists. The SPPB consists of 3 physical performance tests to assess each frailty domain, including balance (static standing balance), gait speed test (4-m walk time), and weakness (time to complete 5 repeated chair stands)^20^. Each test is scored from 0–4, for a total score of 0–12. For balance, the participants were asked to maintain their feet in side-by-side, semi-tandem, and tandem positions for 10 s each. For the gait speed assessment test, the participants’ usual speed was timed during a 4-m walk. For the chair stand test, participants were asked to stand up and sit down five times as quickly as possible. The 6-min walk distance was assessed in an unobstructed hallway according to the guideline as follows^21^: patients were instructed to walk as fast as possible between two points positioned 30-m apart, and the distance walked in 6 min was recorded. Patients were allowed to use an assistive device if needed.

### Assessment of nutritional status

GNRI^22^ was used to evaluate the nutritional status of patients. The index was calculated as follows: 14.89 × serum albumin concentration (g/L) + 41.7 × (weight [kg]/ideal weight [kg]). Ideal weight was defined as: 22 × height (m^2^).

### Outcomes

The prognoses of registered patients within 1 year of discharge were prospectively collected up to March 2019. Our primary outcome was all-cause death. After discharge, most patients were followed up in outpatient clinics at least every 3 months, and additionally on a need basis. For those without in-person follow-up scheduled in clinics, prognostic data were obtained from telephone interviews and medical records of other medical departments that cared for the patient or from the family.

### Statistical analysis

Normally distributed data are expressed as mean and standard deviation, and non-normally distributed data are reported as median with interquartile range. Categorical data are shown as numbers and percentages. Data were compared between groups using Student’s t-tests or Mann–Whitney U tests for continuous variables and chi-squared or Fisher exact tests for categorical variables as appropriate. The associations between AAR and physical function and nutritional status were investigated using linear regression analysis. In the multivariable linear regression analyses, age and gender were used as adjustment variables^23–29^. Association between physical frailty and AAR was evaluated with logistic regression analysis. Age, sex and body mass index were used as adjustment variable in the multivariable logistic regression analyses^30^. Regarding time-dependent survival analysis, event-free survival curves were constructed using the Kaplan–Meier survival method and compared with log-rank statistics. The AUC for 1-year mortality was used to evaluate the predictive value of AAR for 1-year mortality. As for the prognostic outcome of all-cause death, the MAGGIC risk score was calculated for each patient as previously described^31^. The discrimination and calibration of this risk score have been well validated in Japanese patients with heart failure^32^. As adding BNP level at discharge has been shown to be associated with improvement of discrimination with adequate calibration^32^, we used the MAGGIC risk score and log-transformed BNP as an adjustment variable in a multivariable prognostic model for the outcome of all-cause death.

A two-tailed *P* value < 0.05 was considered statistically significant. Statistical analyses were performed using R version 3.1.2 (R Foundation for Statistical Computing, Vienna, Austria; ISBN 3-900051-07-0, URL http://www.R-project.org).

## Supplementary Information

Supplementary Tables.

## Data Availability

The datasets generated during and/or analyzed during the current study are available from the corresponding author upon reasonable request.
