# Supplementary material for: Aspartate aminotransferase to alanine aminotransferase ratio is associated with frailty and mortality in older patients with heart failure
Source: Sci Rep. 2021 Jun 7;11:11957. doi: 10.1038/s41598-021-91368-z (PMC8184951; doi:10.1038/s41598-021-91368-z)
Supplement: Supplementary file 1 — Supplementary Tables. [file 41598_2021_91368_MOESM1_ESM.docx]

| Supplementary Table 1. Association between alanine aminotransferase tertiles and physical status | | | | | | | | | | | | | | | | |  |
| --- | --- | --- | --- | --- | --- | --- | --- | --- | --- | --- | --- | --- | --- | --- | --- | --- | --- |
|  | | | | | | | | | | | | | | |  |  |  |
|  |  | Unadjusted model | | | | | | |  | Adjusted model* | | | | | | |  |
|  |  | Coefficient | | Standard error | t value | | P value | |  | Coefficient | | Standard error | t value | | P value | |  |
| Hand grip strength |  |  |  |  |  |  |  |  |  |  |  |  |  |  |  |  |  |
| 1st vs. 2nd ALT |  |  | 2.91 | 0.51 |  | 5.76 | < | 0.001 |  |  | 1.25 | 0.38 |  | 3.30 | < | 0.001 |  |
| 1st vs. 3rd ALT |  |  | 5.97 | 0.50 |  | 11.94 | < | 0.001 |  |  | 1.84 | 0.40 |  | 4.64 | < | 0.001 |  |
| 6-minute walk distance |  |  |  |  |  |  |  |  |  |  |  |  |  |  |  |  |  |
| 1st vs. 2nd ALT |  |  | 31.02 | 8.70 |  | 3.56 | < | 0.001 |  |  | 11.50 | 7.81 |  | 1.47 |  | 0.14 |  |
| 1st vs. 3rd ALT |  |  | 58.36 | 8.57 |  | 6.81 | < | 0.001 |  |  | 12.23 | 8.06 |  | 1.52 |  | 0.13 |  |
| SPPB |  |  |  |  |  |  |  |  |  |  |  |  |  |  |  |  |  |
| 1st vs. 2nd ALT |  |  | 0.72 | 0.21 |  | 3.35 | < | 0.001 |  |  | 0.21 | 0.19 |  | 1.13 |  | 0.26 |  |
| 1st vs. 3rd ALT |  |  | 1.51 | 0.21 |  | 7.13 | < | 0.001 |  |  | 0.32 | 0.20 |  | 1.62 |  | 0.11 |  |
| GNRI |  |  |  |  |  |  |  |  |  |  |  |  |  |  |  |  |  |
| 1st vs. 2nd ALT |  |  | 3.56 | 0.74 |  | 4.81 | < | 0.001 |  |  | 2.95 | 0.72 |  | 4.08 | < | 0.001 |  |
| 1st vs. 3rd ALT |  |  | 3.88 | 0.73 |  | 5.34 | < | 0.001 |  |  | 2.38 | 0.75 |  | 3.19 |  | 0.002 |  |
|  |  |  |  |  |  |  |  |  |  |  |  |  |  |  |  |  |  |
|  |  |  |  |  |  |  |  |  |  |  |  |  |  |  |  |  |  |
| ALT, alanine aminotransferase; SPPB, short physical performance battery; GNRI, geriatric nutritional risk index | | | | | | | | | | | | | | | | |  |
|  |  |  |  |  |  |  |  |  |  |  |  |  |  |  |  |  |  |
| * Adjusted for age and sex. | | | | | | | | | | | | | | | | |  |
|  |  |  |  |  |  |  |  |  |  |  |  |  |  |  |  |  |  |

| Supplementary Table 2. Association between ALT tertile and physical status | | | | | | | | | | | | | | | | |
| --- | --- | --- | --- | --- | --- | --- | --- | --- | --- | --- | --- | --- | --- | --- | --- | --- |
|  | | | | | | | | | | | | | | |  |  |
|  |  | Unadjusted model | | | | | | |  | Adjusted model* | | | | | | |
|  |  | Coefficient | | Standard error | t value | | P value | |  | Coefficient | | Standard error | t value | | P value | |
| Hand grip strength |  |  |  |  |  |  |  |  |  |  |  |  |  |  |  |  |
| 1st vs. 2nd AST |  |  | 1.41 | 0.53 |  | 2.68 |  | 0.007 |  |  | 0.82 | 0.38 |  | 2.16 |  | 0.031 |
| 1st vs. 3rd AST |  |  | 2.88 | 0.53 |  | 5.45 | < | 0.001 |  |  | 0.74 | 0.39 |  | 1.91 |  | 0.056 |
| 6-minute walk distance |  |  |  |  |  |  |  |  |  |  |  |  |  |  |  |  |
| 1st vs. 2nd AST |  |  | 5.48 | 8.82 |  | 0.62 |  | 0.53 |  |  | 1.00 | 7.74 |  | 0.13 |  | 0.90 |
| 1st vs. 3rd AST |  |  | 25.94 | 8.85 |  | 2.93 |  | 0.003 |  |  | 3.02 | 7.86 |  | 0.38 |  | 0.70 |
| SPPB |  |  |  |  |  |  |  |  |  |  |  |  |  |  |  |  |
| 1st vs. 2nd AST |  |  | 0.26 | 0.22 |  | 1.19 |  | 0.23 |  |  | 0.20 | 0.19 |  | 1.08 |  | 0.28 |
| 1st vs. 3rd AST |  |  | 0.78 | 0.22 |  | 3.61 | < | 0.001 |  |  | 0.20 | 0.19 |  | 1.06 |  | 0.29 |
| GNRI |  |  |  |  |  |  |  |  |  |  |  |  |  |  |  |  |
| 1st vs. 2nd AST |  |  | 1.65 | 0.75 |  | 2.22 |  | 0.027 |  |  | 1.70 | 0.72 |  | 2.36 |  | 0.019 |
| 1st vs. 3rd AST |  |  | 1.43 | 0.75 |  | 1.92 |  | 0.055 |  |  | 0.58 | 0.73 |  | 0.8 |  | 0.43 |
|  |  |  |  |  |  |  |  |  |  |  |  |  |  |  |  |  |
| * Adjusted for age and gender. | | | | | | | | | | | | | | | | |
| ALT, alanine aminotransferase; GNRI, geriatric nutritional risk index; SPPB, short physical performance battery | | | | | | | | | | | | | | | | |

| Supplementary Table 3. Cox proportional hazard analysis for all-cause death according to aspartate aminotransferase and alanine aminotransferase ratio tertiles | | | | | | | | | | | | | | |  |
| --- | --- | --- | --- | --- | --- | --- | --- | --- | --- | --- | --- | --- | --- | --- | --- |
|  |  |  |  |  |  |  |  |  |  |  |  |  |  |  |  |
|  | | | | | | | | | | |  |  |  |  |  |
|  |  | Unadjusted model | | | | | |  | Adjusted model* | | | | | |  |
|  |  | Hazard ratio | 95% CI | | |  | P value |  | Hazard ratio | 95% CI | | |  | P value |  |
| AST tertile |  |  |  |  |  |  |  |  |  |  |  |  |  |  |  |
| 1st AST |  | 1 (reference) | |  |  |  |  |  | 1 (reference) | |  |  |  |  |  |
| 2nd AST |  | 0.84 | 0.57 | – | 1.24 |  | 0.38 |  | 1.04 | 0.68 | – | 1.59 |  | 0.86 |  |
| 3rd AST |  | 1.02 | 0.70 | – | 1.48 |  | 0.92 |  | 1.30 | 0.87 | – | 1.94 |  | 0.21 |  |
|  |  |  |  |  |  |  |  |  |  |  |  |  |  |  |  |
| ALT tertile |  |  |  |  |  |  |  |  |  |  |  |  |  |  |  |
| 1st ALT |  | 1 (reference) | |  |  |  |  |  | 1 (reference) | |  |  |  |  |  |
| 2nd ALT |  | 0.61 | 0.42 | – | 0.90 |  | 0.012 |  | 0.80 | 0.53 | – | 1.21 |  | 0.28 |  |
| 3rd ALT |  | 0.61 | 0.42 | – | 0.90 |  | 0.012 |  | 0.87 | 0.58 | – | 1.31 |  | 0.50 |  |
|  |  |  |  |  |  |  |  |  |  |  |  |  |  |  |  |
| CI, confidence interval; AST, aspartate aminotransferase; ALT, alanine aminotransferase | | | | | | | | | | | | | | |  |
| * Adjusted for Meta-Analysis Global Group in Chronic Heart Failure risk score and log-transformed brain natriuretic peptide. | | | | | | | | | | | | | | |  |
